# Inhalation in the Treatment of Disease

**Published:** 1876-10

**Authors:** 


*Inhalation in the Treatment of Disease: Its Therapeutics and Practice. A treatise on the inhalation of gases, vapors, fumes, compressed and rarified air, nebulized fluids, and powders.* By J. Solis Cohen, M. D., etc. Second Revised Edition. Philadelphia: Lindsay and Blackiston, 1876. Buffalo: Theo. Butler & Son.

Dr. Cohen has divided this work into four parts or sections. The first treats of the inhalation of airs, gases, vapors and fumes; the second considers the use of nebulized vapors or sprays; the third of the inhalation of powders; the fourth considers medicated atmospheres. A large share of the stock in trade of quacks and charlatans has been the use of inhalations of various kinds, and for this reason the profession have come to look upon their employment with disfavor, and have been prejudiced against giving them even a fair amount of study and investigation. Dr. Cohen has brought to the task which he has undertaken in the preparation of this work, an excellent knowledge of the diseases generally treated by inhalations and the results obtained in those affections by other forms of treatment. He has, by careful research, drawn from the published experience of all writers of importance upon this topic, and given to the profession as the result a work which will be of great value to them in the treatment of certain of the diseases of the air passages. The work is amply illustrated, and the explanations concerning the various modes of employing inhalations are sufficiently explicit for all. Dr. Cohen has given a work to the profession which will be of much value.

